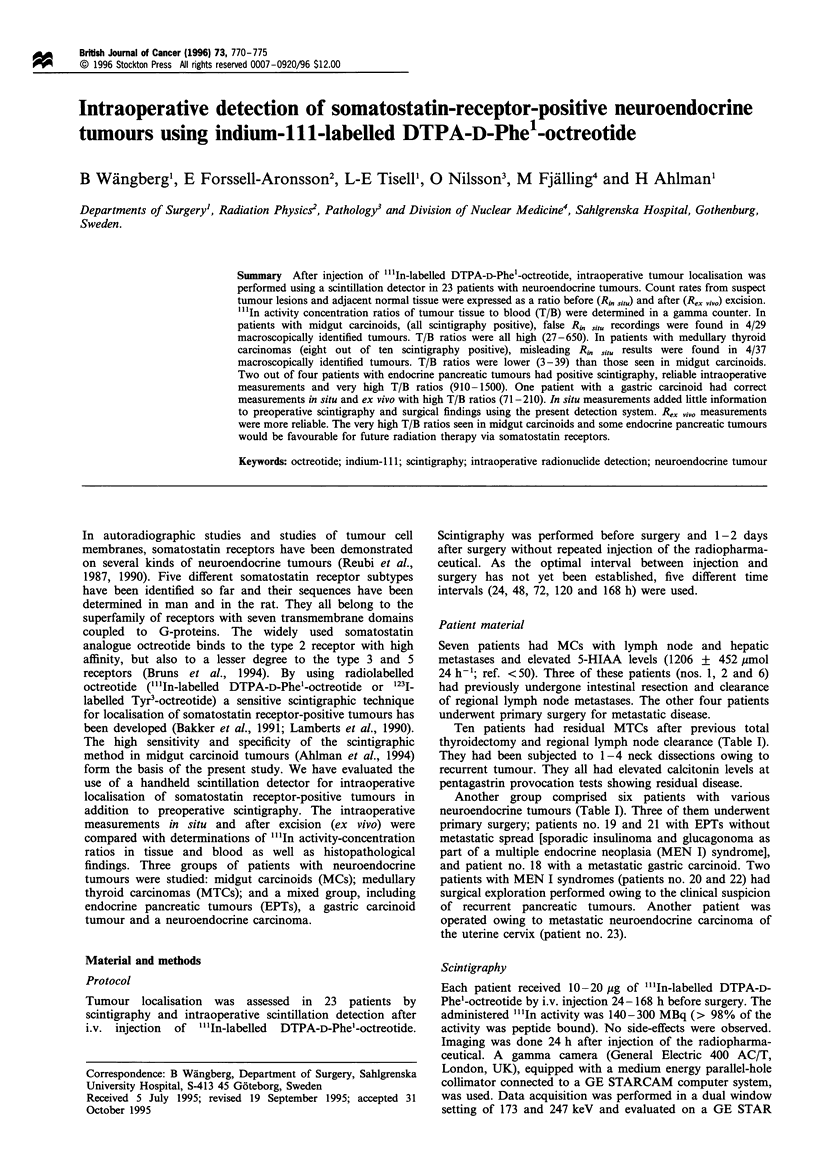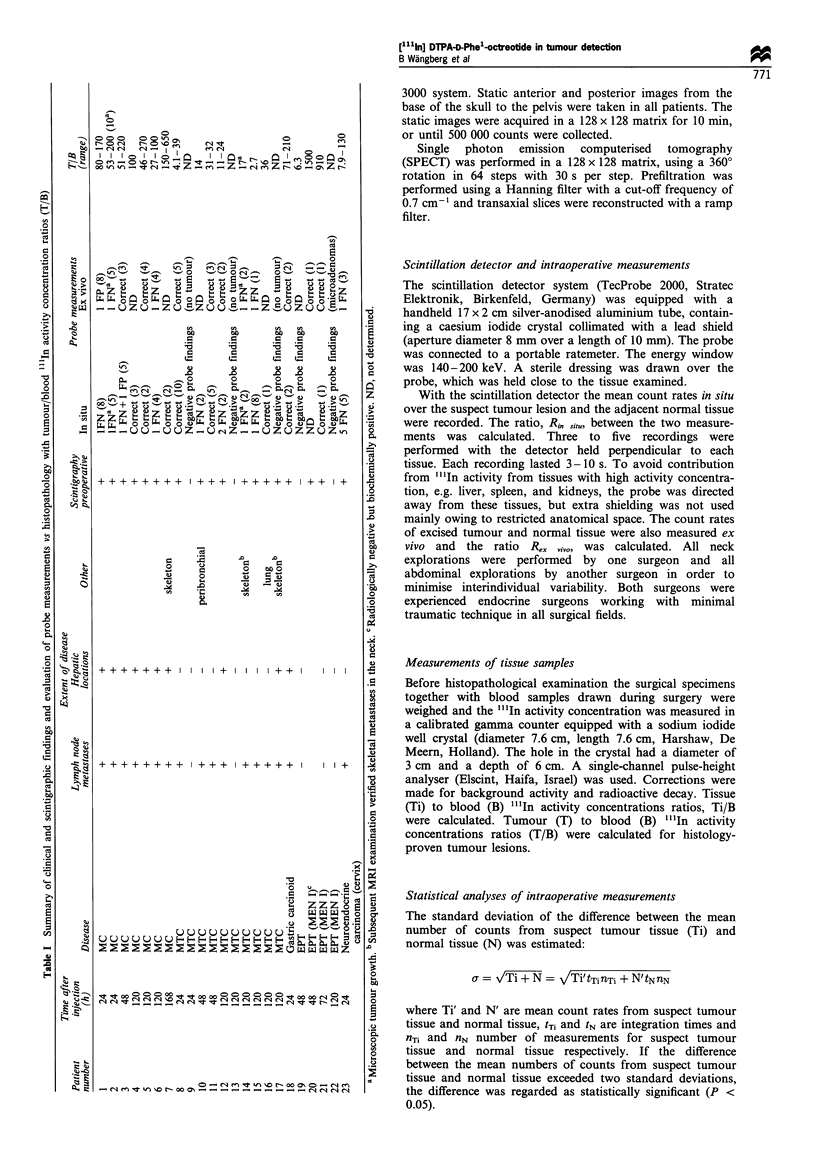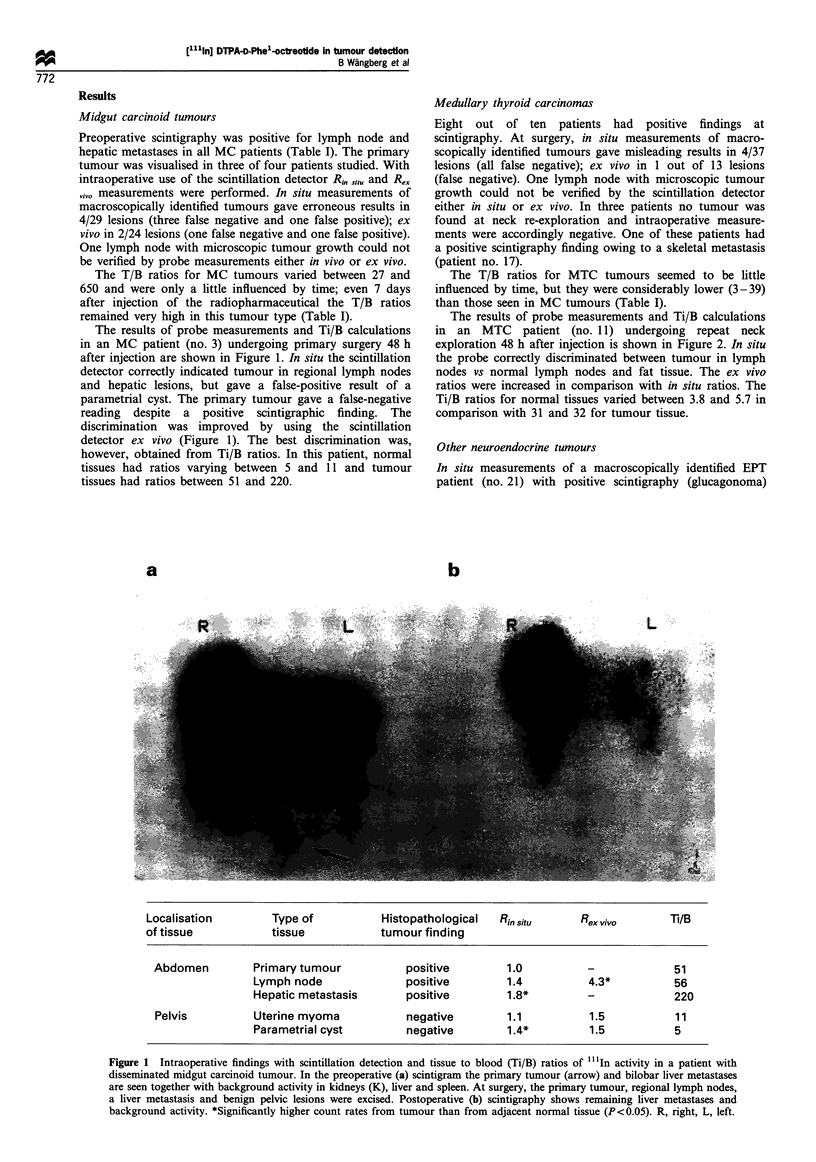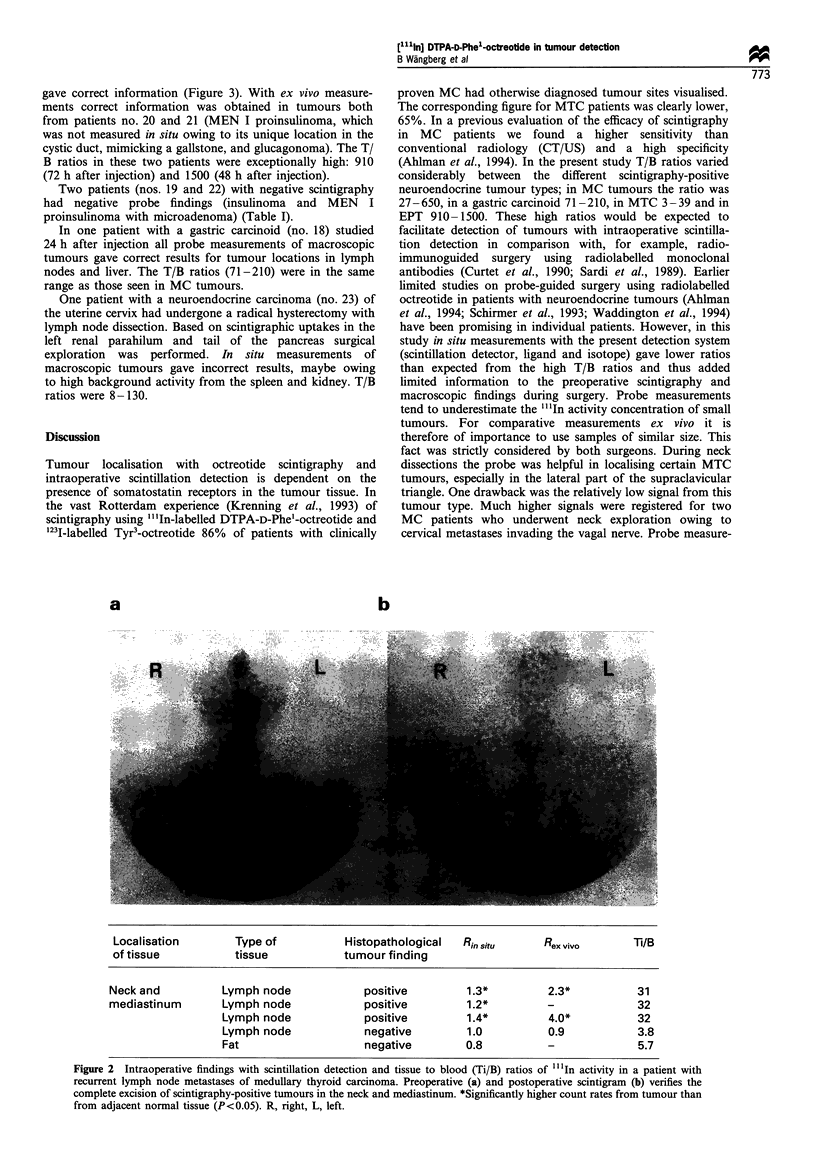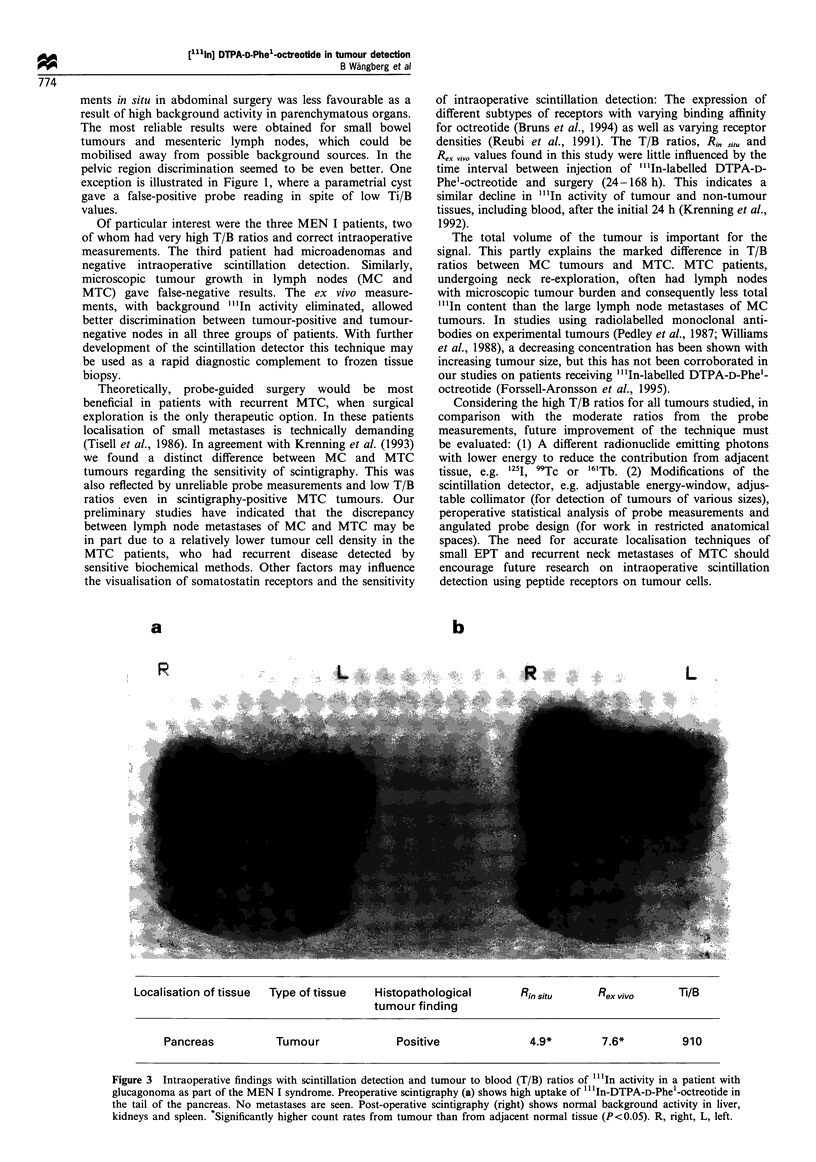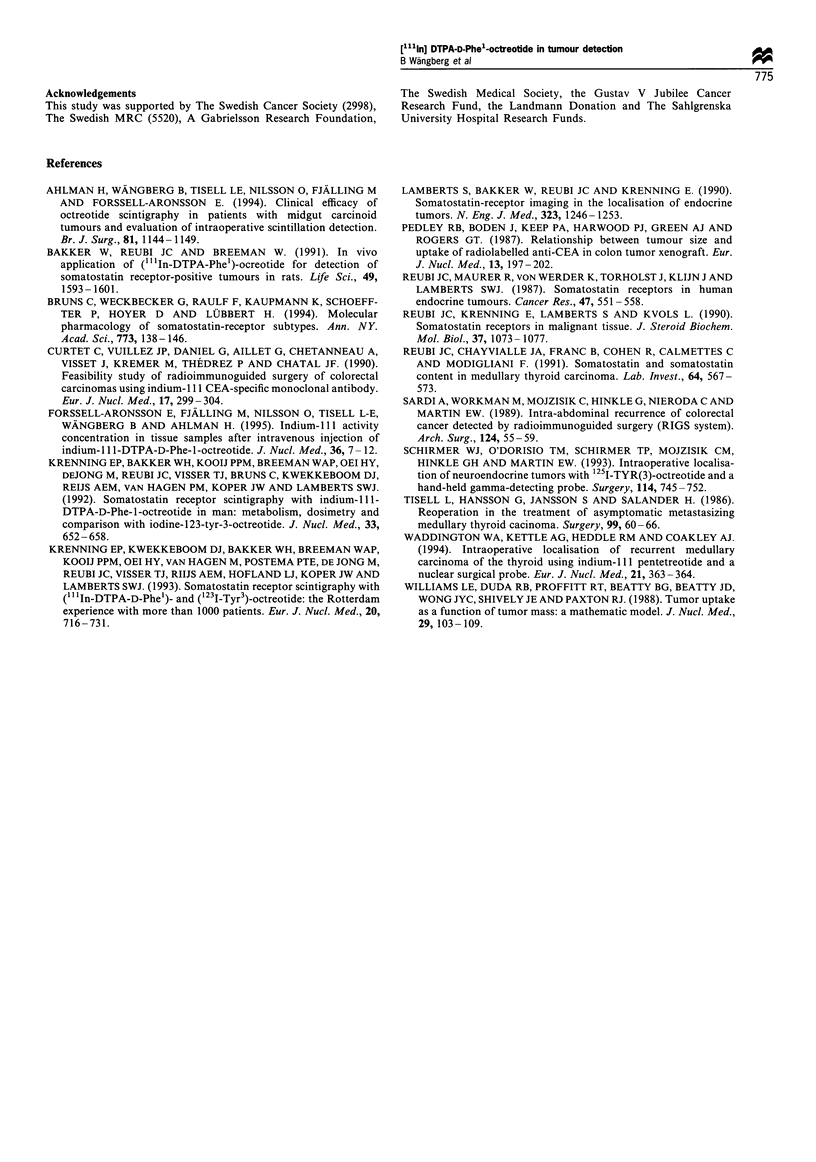# Intraoperative detection of somatostatin-receptor-positive neuroendocrine tumours using indium-111-labelled DTPA-D-Phe1-octreotide.

**DOI:** 10.1038/bjc.1996.134

**Published:** 1996-03

**Authors:** B. Wangberg, E. Forssell-Aronsson, L. E. Tisell, O. Nilsson, M. Fjalling, H. Ahlman

**Affiliations:** Department of Surgery, Sahlgrenska Hospital, Gothenburg, Sweden.

## Abstract

**Images:**


British Journal of Cancer (1996) 73, 770-775
© 1996 Stockton Press All rights reserved 0007-0920/96 $12.00

### Intraoperative detection of somatostatin-receptor-positive neuroendocrine tumours using indium-111-labelled DTPA-D-Phe[1]-octreotide

B Wängberg[1], E Forssell-Aronsson[2], L-E Tisell[1], O Nilsson[3], M Fjälling[4] and H Ahlman[1]

*Departments of Surgery[1], Radiation Physics[2], Pathology[3] and Division of Nuclear Medicine[4], Sahlgrenska Hospital, Gothenburg, Sweden.*

**Summary** After injection of $^{111}$In-labelled DTPA-D-Phe[1]-octreotide, intraoperative tumour localisation was performed using a scintillation detector in 23 patients with neuroendocrine tumours. Count rates from suspect tumour lesions and adjacent normal tissue were expressed as a ratio before ($R_{in\ situ}$) and after ($R_{ex\ vivo}$) excision. $^{111}$In activity concentration ratios of tumour tissue to blood (T/B) were determined in a gamma counter. In patients with midgut carcinoids, (all scintigraphy positive), false $R_{in\ situ}$ recordings were found in 4/29 macroscopically identified tumours. T/B ratios were all high (27-650). In patients with medullary thyroid carcinomas (eight out of ten scintigraphy positive), misleading $R_{in\ situ}$ results were found in 4/37 macroscopically identified tumours. T/B ratios were lower (3-39) than those seen in midgut carcinoids. Two out of four patients with endocrine pancreatic tumours had positive scintigraphy, reliable intraoperative measurements and very high T/B ratios (910-1500). One patient with a gastric carcinoid had correct measurements *in situ* and *ex vivo* with high T/B ratios (71-210). *In situ* measurements added little information to preoperative scintigraphy and surgical findings using the present detection system. $R_{ex\ vivo}$ measurements were more reliable. The very high T/B ratios seen in midgut carcinoids and some endocrine pancreatic tumours would be favourable for future radiation therapy via somatostatin receptors.

**Keywords:** octreotide; indium-111; scintigraphy; intraoperative radionuclide detection; neuroendocrine tumour

In autoradiographic studies and studies of tumour cell membranes, somatostatin receptors have been demonstrated on several kinds of neuroendocrine tumours (Reubi *et al.*, 1987, 1990). Five different somatostatin receptor subtypes have been identified so far and their sequences have been determined in man and in the rat. They all belong to the superfamily of receptors with seven transmembrane domains coupled to G-proteins. The widely used somatostatin analogue octreotide binds to the type 2 receptor with high affinity, but also to a lesser degree to the type 3 and 5 receptors (Bruns *et al.*, 1994). By using radiolabelled octreotide ($^{111}$In-labelled DTPA-D-Phe[1]-octreotide or $^{123}$I-labelled Tyr[3]-octreotide) a sensitive scintigraphic technique for localisation of somatostatin receptor-positive tumours has been developed (Bakker *et al.*, 1991; Lamberts *et al.*, 1990). The high sensitivity and specificity of the scintigraphic method in midgut carcinoid tumours (Ahlman *et al.*, 1994) form the basis of the present study. We have evaluated the use of a handheld scintillation detector for intraoperative localisation of somatostatin receptor-positive tumours in addition to preoperative scintigraphy. The intraoperative measurements *in situ* and after excision (*ex vivo*) were compared with determinations of $^{111}$In activity-concentration ratios in tissue and blood as well as histopathological findings. Three groups of patients with neuroendocrine tumours were studied: midgut carcinoids (MCs); medullary thyroid carcinomas (MTCs); and a mixed group, including endocrine pancreatic tumours (EPTs), a gastric carcinoid tumour and a neuroendocrine carcinoma.

### Material and methods

### Protocol

Tumour localisation was assessed in 23 patients by scintigraphy and intraoperative scintillation detection after i.v. injection of $^{111}$In-labelled DTPA-D-Phe[1]-octreotide. Scintigraphy was performed before surgery and 1-2 days after surgery without repeated injection of the radiopharmaceutical. As the optimal interval between injection and surgery has not yet been established, five different time intervals (24, 48, 72, 120 and 168 h) were used.

### Patient material

Seven patients had MCs with lymph node and hepatic metastases and elevated 5-HIAA levels (1206 ± 452 $\mu$mol 24 h$^{-1}$; ref. <50). Three of these patients (nos. 1, 2 and 6) had previously undergone intestinal resection and clearance of regional lymph node metastases. The other four patients underwent primary surgery for metastatic disease.

Ten patients had residual MTCs after previous total thyroidectomy and regional lymph node clearance (Table I). They had been subjected to 1-4 neck dissections owing to recurrent tumour. They all had elevated calcitonin levels at pentagastrin provocation tests showing residual disease.

Another group comprised six patients with various neuroendocrine tumours (Table I). Three of them underwent primary surgery; patients no. 19 and 21 with EPTs without metastatic spread [sporadic insulinoma and glucagonoma as part of a multiple endocrine neoplasia (MEN I) syndrome], and patient no. 18 with a metastatic gastric carcinoid. Two patients with MEN I syndromes (patients no. 20 and 22) had surgical exploration performed owing to the clinical suspicion of recurrent pancreatic tumours. Another patient was operated owing to metastatic neuroendocrine carcinoma of the uterine cervix (patient no. 23).

### Scintigraphy

Each patient received 10-20 $\mu$g of $^{111}$In-labelled DTPA-D-Phe[1]-octreotide by i.v. injection 24-168 h before surgery. The administered $^{111}$In activity was 140-300 MBq (> 98% of the activity was peptide bound). No side-effects were observed. Imaging was done 24 h after injection of the radiopharmaceutical. A gamma camera (General Electric 400 AC/T, London, UK), equipped with a medium energy parallel-hole collimator connected to a GE STARCAM computer system, was used. Data acquisition was performed in a dual window setting of 173 and 247 keV and evaluated on a GE STAR

Correspondence: B Wängberg, Department of Surgery, Sahlgrenska University Hospital, S-413 45 Göteborg, Sweden
Received 5 July 1995; revised 19 September 1995; accepted 31 October 1995

**Table I** Summary of clinical and scintigraphic findings and evaluation of probe measurements *vs* histopathology with tumour/blood $^{111}$In activity concentration ratios (T/B)

| *Patient number* | *Time after injection (h)* | *Disease* | *Extent of disease* Lymph node metastases | *Extent of disease* Hepatic locations | *Extent of disease* Other | *Scintigraphy preoperative* | *Probe measurements* In situ | *Probe measurements* Ex vivo | *T/B (range)* |
|---|---|---|---|---|---|---|---|---|---|
| 1 | 24 | MC | + | + | | + | 1FN (8) | 1 FP (8) | 80–170 |
| 2 | 24 | MC | + | + | | + | 1FN[a] (5) | 1 FN[a] (5) | 53–200 (10[a]) |
| 3 | 48 | MC | + | + | | + | 1 FN+1 FP (5) | Correct (3) | 51–220 |
| 4 | 120 | MC | + | + | | + | Correct (3) | ND | 100 |
| 5 | 120 | MC | + | + | | + | Correct (2) | Correct (4) | 46–270 |
| 6 | 120 | MC | + | + | | + | 1 FN (4) | 1 FN (4) | 27–100 |
| 7 | 168 | MC | + | + | skeleton | + | Correct (2) | ND | 150–650 |
| 8 | 24 | MTC | + | – | | + | Correct (10) | Correct (5) | 4.1–39 |
| 9 | 24 | MTC | – | – | | – | Negative probe findings | (no tumour) | ND |
| 10 | 48 | MTC | + | – | peribronchial | + | 1 FN (2) | ND | 14 |
| 11 | 48 | MTC | + | – | | + | Correct (5) | Correct (3) | 31–32 |
| 12 | 120 | MTC | + | + | | + | 2 FN (2) | Correct (2) | 11–24 |
| 13 | 120 | MTC | – | – | | – | Negative probe findings | (no tumour) | ND |
| 14 | 120 | MTC | + | – | skeleton[b] | + | 1 FN[a] (2) | 1 FN[a] (2) | 17[a] |
| 15 | 120 | MTC | + | – | | + | 1 FN (8) | 1 FN (1) | 2.7 |
| 16 | 120 | MTC | + | – | lung | + | Correct (1) | ND | 36 |
| 17 | 120 | MTC | + | + | skeleton[b] | + | Negative probe findings | (no tumour) | ND |
| 18 | 24 | Gastric carcinoid | + | + | | + | Correct (2) | Correct (2) | 71–210 |
| 19 | 48 | EPT | – | – | | – | Negative probe findings | ND | 6.3 |
| 20 | 48 | EPT (MEN I)[c] | | | | + | ND | Correct (1) | 1500 |
| 21 | 72 | EPT (MEN I) | – | – | | + | Correct (1) | Correct (1) | 910 |
| 22 | 120 | EPT (MEN I) | – | – | | – | Negative probe findings | (microadenomas) | ND |
| 23 | 24 | Neuroendocrine carcinoma (cervix) | + | – | | + | 5 FN (5) | 1 FN (3) | 7.9–130 |

[a] Microscopic tumour growth. [b] Subsequent MRI examination verified skeletal metastases in the neck. [c] Radiologically negative but biochemically positive. ND, not determined.

3000 system. Static anterior and posterior images from the base of the skull to the pelvis were taken in all patients. The static images were acquired in a 128 × 128 matrix for 10 min, or until 500 000 counts were collected.

Single photon emission computerised tomography (SPECT) was performed in a 128 × 128 matrix, using a 360° rotation in 64 steps with 30 s per step. Prefiltration was performed using a Hanning filter with a cut-off frequency of 0.7 $cm^{-1}$ and transaxial slices were reconstructed with a ramp filter.

### *Scintillation detector and intraoperative measurements*

The scintillation detector system (TecProbe 2000, Stratec Elektronik, Birkenfeld, Germany) was equipped with a handheld 17 × 2 cm silver-anodised aluminium tube, containing a caesium iodide crystal collimated with a lead shield (aperture diameter 8 mm over a length of 10 mm). The probe was connected to a portable ratemeter. The energy window was 140–200 keV. A sterile dressing was drawn over the probe, which was held close to the tissue examined.

With the scintillation detector the mean count rates *in situ* over the suspect tumour lesion and the adjacent normal tissue were recorded. The ratio, $R_{in\ situ}$, between the two measurements was calculated. Three to five recordings were performed with the detector held perpendicular to each tissue. Each recording lasted 3–10 s. To avoid contribution from $^{111}$In activity from tissues with high activity concentration, e.g. liver, spleen, and kidneys, the probe was directed away from these tissues, but extra shielding was not used mainly owing to restricted anatomical space. The count rates of excised tumour and normal tissue were also measured *ex vivo* and the ratio $R_{ex\ vivo}$, was calculated. All neck explorations were performed by one surgeon and all abdominal explorations by another surgeon in order to minimise interindividual variability. Both surgeons were experienced endocrine surgeons working with minimal traumatic technique in all surgical fields.

### *Measurements of tissue samples*

Before histopathological examination the surgical specimens together with blood samples drawn during surgery were weighed and the $^{111}$In activity concentration was measured in a calibrated gamma counter equipped with a sodium iodide well crystal (diameter 7.6 cm, length 7.6 cm, Harshaw, De Meern, Holland). The hole in the crystal had a diameter of 3 cm and a depth of 6 cm. A single-channel pulse-height analyser (Elscint, Haifa, Israel) was used. Corrections were made for background activity and radioactive decay. Tissue (Ti) to blood (B) $^{111}$In activity concentrations ratios, Ti/B were calculated. Tumour (T) to blood (B) $^{111}$In activity concentrations ratios (T/B) were calculated for histology-proven tumour lesions.

### *Statistical analyses of intraoperative measurements*

The standard deviation of the difference between the mean number of counts from suspect tumour tissue (Ti) and normal tissue (N) was estimated:

$$\sigma = \sqrt{\mathrm{Ti} + \mathrm{N}} = \sqrt{\mathrm{Ti}' t_{\mathrm{Ti}} n_{\mathrm{Ti}} + \mathrm{N}' t_{\mathrm{N}} n_{\mathrm{N}}}$$

where Ti′ and N′ are mean count rates from suspect tumour tissue and normal tissue, $t_{Ti}$ and $t_N$ are integration times and $n_{Ti}$ and $n_N$ number of measurements for suspect tumour tissue and normal tissue respectively. If the difference between the mean numbers of counts from suspect tumour tissue and normal tissue exceeded two standard deviations, the difference was regarded as statistically significant ($P <$ 0.05).

### Results

### *Midgut carcinoid tumours*

Preoperative scintigraphy was positive for lymph node and hepatic metastases in all MC patients (Table I). The primary tumour was visualised in three of four patients studied. With intraoperative use of the scintillation detector $R_{in\ situ}$ and $R_{ex\ vivo}$ measurements were performed. *In situ* measurements of macroscopically identified tumours gave erroneous results in 4/29 lesions (three false negative and one false positive); *ex vivo* in 2/24 lesions (one false negative and one false positive). One lymph node with microscopic tumour growth could not be verified by probe measurements either *in vivo* or *ex vivo*.

The T/B ratios for MC tumours varied between 27 and 650 and were only a little influenced by time; even 7 days after injection of the radiopharmaceutical the T/B ratios remained very high in this tumour type (Table I).

The results of probe measurements and Ti/B calculations in an MC patient (no. 3) undergoing primary surgery 48 h after injection are shown in Figure 1. *In situ* the scintillation detector correctly indicated tumour in regional lymph nodes and hepatic lesions, but gave a false-positive result of a parametrial cyst. The primary tumour gave a false-negative reading despite a positive scintigraphic finding. The discrimination was improved by using the scintillation detector *ex vivo* (Figure 1). The best discrimination was, however, obtained from Ti/B ratios. In this patient, normal tissues had ratios varying between 5 and 11 and tumour tissues had ratios between 51 and 220.

### *Medullary thyroid carcinomas*

Eight out of ten patients had positive findings at scintigraphy. At surgery, *in situ* measurements of macroscopically identified tumours gave misleading results in 4/37 lesions (all false negative); *ex vivo* in 1 out of 13 lesions (false negative). One lymph node with microscopic tumour growth could not be verified by the scintillation detector either *in situ* or *ex vivo*. In three patients no tumour was found at neck re-exploration and intraoperative measurements were accordingly negative. One of these patients had a positive scintigraphy finding owing to a skeletal metastasis (patient no. 17).

The T/B ratios for MTC tumours seemed to be little influenced by time, but they were considerably lower (3–39) than those seen in MC tumours (Table I).

The results of probe measurements and Ti/B calculations in an MTC patient (no. 11) undergoing repeat neck exploration 48 h after injection is shown in Figure 2. *In situ* the probe correctly discriminated between tumour in lymph nodes *vs* normal lymph nodes and fat tissue. The *ex vivo* ratios were increased in comparison with *in situ* ratios. The Ti/B ratios for normal tissues varied between 3.8 and 5.7 in comparison with 31 and 32 for tumour tissue.

### *Other neuroendocrine tumours*

*In situ* measurements of a macroscopically identified EPT patient (no. 21) with positive scintigraphy (glucagonoma)

| Localisation of tissue | Type of tissue | Histopathological tumour finding | $R_{in\ situ}$ | $R_{ex\ vivo}$ | Ti/B |
|---|---|---|---|---|---|
| Abdomen | Primary tumour | positive | 1.0 | – | 51 |
| | Lymph node | positive | 1.4 | 4.3* | 56 |
| | Hepatic metastasis | positive | 1.8* | – | 220 |
| Pelvis | Uterine myoma | negative | 1.1 | 1.5 | 11 |
| | Parametrial cyst | negative | 1.4* | 1.5 | 5 |

**Figure 1** Intraoperative findings with scintillation detection and tissue to blood (Ti/B) ratios of $^{111}$In activity in a patient with disseminated midgut carcinoid tumour. In the preoperative (**a**) scintigram the primary tumour (arrow) and bilobar liver metastases are seen together with background activity in kidneys (K), liver and spleen. At surgery, the primary tumour, regional lymph nodes, a liver metastasis and benign pelvic lesions were excised. Postoperative (**b**) scintigraphy shows remaining liver metastases and background activity. *Significantly higher count rates from tumour than from adjacent normal tissue ($P<0.05$). R, right, L, left.

gave correct information (Figure 3). With *ex vivo* measurements correct information was obtained in tumours both from patients no. 20 and 21 (MEN I proinsulinoma, which was not measured *in situ* owing to its unique location in the cystic duct, mimicking a gallstone, and glucagonoma). The T/B ratios in these two patients were exceptionally high: 910 (72 h after injection) and 1500 (48 h after injection).

Two patients (nos. 19 and 22) with negative scintigraphy had negative probe findings (insulinoma and MEN I proinsulinoma with microadenoma) (Table I).

In one patient with a gastric carcinoid (no. 18) studied 24 h after injection all probe measurements of macroscopic tumours gave correct results for tumour locations in lymph nodes and liver. The T/B ratios (71–210) were in the same range as those seen in MC tumours.

One patient with a neuroendocrine carcinoma (no. 23) of the uterine cervix had undergone a radical hysterectomy with lymph node dissection. Based on scintigraphic uptakes in the left renal parahilum and tail of the pancreas surgical exploration was performed. *In situ* measurements of macroscopic tumours gave incorrect results, maybe owing to high background activity from the spleen and kidney. T/B ratios were 8–130.

### Discussion

Tumour localisation with octreotide scintigraphy and intraoperative scintillation detection is dependent on the presence of somatostatin receptors in the tumour tissue. In the vast Rotterdam experience (Krenning *et al.*, 1993) of scintigraphy using $^{111}$In-labelled DTPA-D-Phe$^1$-octreotide and $^{123}$I-labelled Tyr$^3$-octreotide 86% of patients with clinically proven MC had otherwise diagnosed tumour sites visualised. The corresponding figure for MTC patients was clearly lower, 65%. In a previous evaluation of the efficacy of scintigraphy in MC patients we found a higher sensitivity than conventional radiology (CT/US) and a high specificity (Ahlman *et al.*, 1994). In the present study T/B ratios varied considerably between the different scintigraphy-positive neuroendocrine tumour types; in MC tumours the ratio was 27–650, in a gastric carcinoid 71–210, in MTC 3–39 and in EPT 910–1500. These high ratios would be expected to facilitate detection of tumours with intraoperative scintillation detection in comparison with, for example, radioimmunoguided surgery using radiolabelled monoclonal antibodies (Curtet *et al.*, 1990; Sardi *et al.*, 1989). Earlier limited studies on probe-guided surgery using radiolabelled octreotide in patients with neuroendocrine tumours (Ahlman *et al.*, 1994; Schirmer *et al.*, 1993; Waddington *et al.*, 1994) have been promising in individual patients. However, in this study *in situ* measurements with the present detection system (scintillation detector, ligand and isotope) gave lower ratios than expected from the high T/B ratios and thus added limited information to the preoperative scintigraphy and macroscopic findings during surgery. Probe measurements tend to underestimate the $^{111}$In activity concentration of small tumours. For comparative measurements *ex vivo* it is therefore of importance to use samples of similar size. This fact was strictly considered by both surgeons. During neck dissections the probe was helpful in localising certain MTC tumours, especially in the lateral part of the supraclavicular triangle. One drawback was the relatively low signal from this tumour type. Much higher signals were registered for two MC patients who underwent neck exploration owing to cervical metastases invading the vagal nerve. Probe measure-

| Localisation of tissue | Type of tissue | Histopathological tumour finding | $R_{in\ situ}$ | $R_{ex\ vivo}$ | Ti/B |
|---|---|---|---|---|---|
| Neck and mediastinum | Lymph node | positive | 1.3* | 2.3* | 31 |
| | Lymph node | positive | 1.2* | – | 32 |
| | Lymph node | positive | 1.4* | 4.0* | 32 |
| | Lymph node | negative | 1.0 | 0.9 | 3.8 |
| | Fat | negative | 0.8 | – | 5.7 |

**Figure 2** Intraoperative findings with scintillation detection and tissue to blood (Ti/B) ratios of $^{111}$In activity in a patient with recurrent lymph node metastases of medullary thyroid carcinoma. Preoperative (**a**) and postoperative scintigram (**b**) verifies the complete excision of scintigraphy-positive tumours in the neck and mediastinum. *Significantly higher count rates from tumour than from adjacent normal tissue ($P<0.05$). R, right, L, left.

ments *in situ* in abdominal surgery was less favourable as a result of high background activity in parenchymatous organs. The most reliable results were obtained for small bowel tumours and mesenteric lymph nodes, which could be mobilised away from possible background sources. In the pelvic region discrimination seemed to be even better. One exception is illustrated in Figure 1, where a parametrial cyst gave a false-positive probe reading in spite of low Ti/B values.

Of particular interest were the three MEN I patients, two of whom had very high T/B ratios and correct intraoperative measurements. The third patient had microadenomas and negative intraoperative scintillation detection. Similarly, microscopic tumour growth in lymph nodes (MC and MTC) gave false-negative results. The *ex vivo* measurements, with background $^{111}$In activity eliminated, allowed better discrimination between tumour-positive and tumour-negative nodes in all three groups of patients. With further development of the scintillation detector this technique may be used as a rapid diagnostic complement to frozen tissue biopsy.

Theoretically, probe-guided surgery would be most beneficial in patients with recurrent MTC, when surgical exploration is the only therapeutic option. In these patients localisation of small metastases is technically demanding (Tisell *et al.*, 1986). In agreement with Krenning *et al.* (1993) we found a distinct difference between MC and MTC tumours regarding the sensitivity of scintigraphy. This was also reflected by unreliable probe measurements and low T/B ratios even in scintigraphy-positive MTC tumours. Our preliminary studies have indicated that the discrepancy between lymph node metastases of MC and MTC may be in part due to a relatively lower tumour cell density in the MTC patients, who had recurrent disease detected by sensitive biochemical methods. Other factors may influence the visualisation of somatostatin receptors and the sensitivity of intraoperative scintillation detection: The expression of different subtypes of receptors with varying binding affinity for octreotide (Bruns *et al.*, 1994) as well as varying receptor densities (Reubi *et al.*, 1991). The T/B ratios, $R_{in\ situ}$ and $R_{ex\ vivo}$ values found in this study were little influenced by the time interval between injection of $^{111}$In-labelled DTPA-D-Phe$^1$-octreotide and surgery (24–168 h). This indicates a similar decline in $^{111}$In activity of tumour and non-tumour tissues, including blood, after the initial 24 h (Krenning *et al.*, 1992).

The total volume of the tumour is important for the signal. This partly explains the marked difference in T/B ratios between MC tumours and MTC. MTC patients, undergoing neck re-exploration, often had lymph nodes with microscopic tumour burden and consequently less total $^{111}$In content than the large lymph node metastases of MC tumours. In studies using radiolabelled monoclonal antibodies on experimental tumours (Pedley *et al.*, 1987; Williams *et al.*, 1988), a decreasing concentration has been shown with increasing tumour size, but this has not been corroborated in our studies on patients receiving $^{111}$In-labelled DTPA-D-Phe$^1$-octreotide (Forssell-Aronsson *et al.*, 1995).

Considering the high T/B ratios for all tumours studied, in comparison with the moderate ratios from the probe measurements, future improvement of the technique must be evaluated: (1) A different radionuclide emitting photons with lower energy to reduce the contribution from adjacent tissue, e.g. $^{125}$I, $^{99}$Tc or $^{161}$Tb. (2) Modifications of the scintillation detector, e.g. adjustable energy-window, adjustable collimator (for detection of tumours of various sizes), peroperative statistical analysis of probe measurements and angulated probe design (for work in restricted anatomical spaces). The need for accurate localisation techniques of small EPT and recurrent neck metastases of MTC should encourage future research on intraoperative scintillation detection using peptide receptors on tumour cells.

| Localisation of tissue | Type of tissue | Histopathological tumour finding | $R_{in\ situ}$ | $R_{ex\ vivo}$ | Ti/B |
|---|---|---|---|---|---|
| Pancreas | Tumour | Positive | 4.9* | 7.6* | 910 |

**Figure 3** Intraoperative findings with scintillation detection and tumour to blood (T/B) ratios of $^{111}$In activity in a patient with glucagonoma as part of the MEN I syndrome. Preoperative scintigraphy (**a**) shows high uptake of $^{111}$In-DTPA-D-Phe$^1$-octreotide in the tail of the pancreas. No metastases are seen. Post-operative scintigraphy (right) shows normal background activity in liver, kidneys and spleen. *Significantly higher count rates from tumour than from adjacent normal tissue ($P<0.05$). R, right, L, left.

**Acknowledgements**
This study was supported by The Swedish Cancer Society (2998), The Swedish MRC (5520), A Gabrielsson Research Foundation, The Swedish Medical Society, the Gustav V Jubilee Cancer Research Fund, the Landmann Donation and The Sahlgrenska University Hospital Research Funds.

### References

AHLMAN H, WÄNGBERG B, TISELL LE, NILSSON O, FJÄLLING M AND FORSSELL-ARONSSON E. (1994). Clinical efficacy of octreotide scintigraphy in patients with midgut carcinoid tumours and evaluation of intraoperative scintillation detection. *Br. J. Surg.*, **81**, 1144–1149.

BAKKER W, REUBI JC AND BREEMAN W. (1991). In vivo application of ($^{111}$In-DTPA-Phe$^1$)-ocreotide for detection of somatostatin receptor-positive tumours in rats. *Life Sci.*, **49**, 1593–1601.

BRUNS C, WECKBECKER G, RAULF F, KAUPMANN K, SCHOEFFTER P, HOYER D AND LÜBBERT H. (1994). Molecular pharmacology of somatostatin-receptor subtypes. *Ann. NY. Acad. Sci.*, **773**, 138–146.

CURTET C, VUILLEZ JP, DANIEL G, AILLET G, CHETANNEAU A, VISSET J, KREMER M, THÉDREZ P AND CHATAL JF. (1990). Feasibility study of radioimmunoguided surgery of colorectal carcinomas using indium-111 CEA-specific monoclonal antibody. *Eur. J. Nucl. Med.*, **17**, 299–304.

FORSSELL-ARONSSON E, FJÄLLING M, NILSSON O, TISELL L-E, WÄNGBERG B AND AHLMAN H. (1995). Indium-111 activity concentration in tissue samples after intravenous injection of indium-111-DTPA-D-Phe-1-octreotide. *J. Nucl. Med.*, **36**, 7–12.

KRENNING EP, BAKKER WH, KOOIJ PPM, BREEMAN WAP, OEI HY, DEJONG M, REUBI JC, VISSER TJ, BRUNS C, KWEKKEBOOM DJ, REIJS AEM, VAN HAGEN PM, KOPER JW AND LAMBERTS SWJ. (1992). Somatostatin receptor scintigraphy with indium-111-DTPA-D-Phe-1-octreotide in man: metabolism, dosimetry and comparison with iodine-123-tyr-3-octreotide. *J. Nucl. Med.*, **33**, 652–658.

KRENNING EP, KWEKKEBOOM DJ, BAKKER WH, BREEMAN WAP, KOOIJ PPM, OEI HY, VAN HAGEN M, POSTEMA PTE, DE JONG M, REUBI JC, VISSER TJ, RIIJS AEM, HOFLAND LJ, KOPER JW AND LAMBERTS SWJ. (1993). Somatostatin receptor scintigraphy with ($^{111}$In-DTPA-D-Phe$^1$)- and ($^{123}$I-Tyr$^3$)-octreotide: the Rotterdam experience with more than 1000 patients. *Eur. J. Nucl. Med.*, **20**, 716–731.

LAMBERTS S, BAKKER W, REUBI JC AND KRENNING E. (1990). Somatostatin-receptor imaging in the localisation of endocrine tumors. *N. Eng. J. Med.*, **323**, 1246–1253.

PEDLEY RB, BODEN J, KEEP PA, HARWOOD PJ, GREEN AJ AND ROGERS GT. (1987). Relationship between tumour size and uptake of radiolabelled anti-CEA in colon tumor xenograft. *Eur. J. Nucl. Med.*, **13**, 197–202.

REUBI JC, MAURER R, VON WERDER K, TORHOLST J, KLIJN J AND LAMBERTS SWJ. (1987). Somatostatin receptors in human endocrine tumours. *Cancer Res.*, **47**, 551–558.

REUBI JC, KRENNING E, LAMBERTS S AND KVOLS L. (1990). Somatostatin receptors in malignant tissue. *J. Steroid Biochem. Mol. Biol.*, **37**, 1073–1077.

REUBI JC, CHAYVIALLE JA, FRANC B, COHEN R, CALMETTES C AND MODIGLIANI F. (1991). Somatostatin and somatostatin content in medullary thyroid carcinoma. *Lab. Invest.*, **64**, 567–573.

SARDI A, WORKMAN M, MOJZISIK C, HINKLE G, NIERODA C AND MARTIN EW. (1989). Intra-abdominal recurrence of colorectal cancer detected by radioimmunoguided surgery (RIGS system). *Arch. Surg.*, **124**, 55–59.

SCHIRMER WJ, O'DORISIO TM, SCHIRMER TP, MOJZISIK CM, HINKLE GH AND MARTIN EW. (1993). Intraoperative localisation of neuroendocrine tumors with $^{125}$I-TYR(3)-octreotide and a hand-held gamma-detecting probe. *Surgery*, **114**, 745–752.

TISELL L, HANSSON G, JANSSON S AND SALANDER H. (1986). Reoperation in the treatment of asymptomatic metastasizing medullary thyroid cacinoma. *Surgery*, **99**, 60–66.

WADDINGTON WA, KETTLE AG, HEDDLE RM AND COAKLEY AJ. (1994). Intraoperative localisation of recurrent medullary carcinoma of the thyroid using indium-111 pentetreotide and a nuclear surgical probe. *Eur. J. Nucl. Med.*, **21**, 363–364.

WILLIAMS LE, DUDA RB, PROFFITT RT, BEATTY BG, BEATTY JD, WONG JYC, SHIVELY JE AND PAXTON RJ. (1988). Tumor uptake as a function of tumor mass: a mathematic model. *J. Nucl. Med.*, **29**, 103–109.